# Read-through Activation of Transcription in a Cellular Genomic Context

**DOI:** 10.1371/journal.pone.0015704

**Published:** 2010-12-28

**Authors:** Li Shen, David J. Spector

**Affiliations:** 1 Department of Microbiology and Immunology, Inter-College Graduate Degree Program in Genetics, College of Medicine, The Pennsylvania State University, Hershey, Pennsylvania, United States of America; 2 Program in Cell and Molecular Biology, College of Medicine, The Pennsylvania State University, Hershey, Pennsylvania, United States of America; Texas A&M University, United States of America

## Abstract

Read-through transcription from the adjacent E1a gene region is required for wild-type (wt) activity of the downstream adenovirus E1b promoter early after infection (read-through activation). However, whether a cellular chromosomal template can support read-through activation is not known. To address this issue, read-through activation was evaluated in the context of stably expressed templates in transfected cells. Inhibition of read-through transcription by insertion of a transcription termination sequence between the E1a and E1b promoters reduced downstream gene expression from stably integrated templates. The results indicate that the mechanism of read-through activation does not depend on the structure of early adenovirus nucleoprotein complexes, a structure that is likely to be different from that of cellular chromatin. Accordingly, this regulatory interaction could participate in the coordinated control of the expression of closely linked cellular genes.

## Introduction

In higher eukaryotic genomes, functionally or developmentally related transcription units are often arranged in groups [1]–[3]. Sometimes, such gene arrangements result in *cis*-acting transcriptional interactions between the genes in a cluster [4]–[8]. One well recognized *cis*-acting transcriptional interaction is transcriptional interference, the suppressive influence of one active transcriptional unit on another linked unit. Transcriptional interference has been described in a variety of experimental systems [9]–[12].

**Figure 1:**
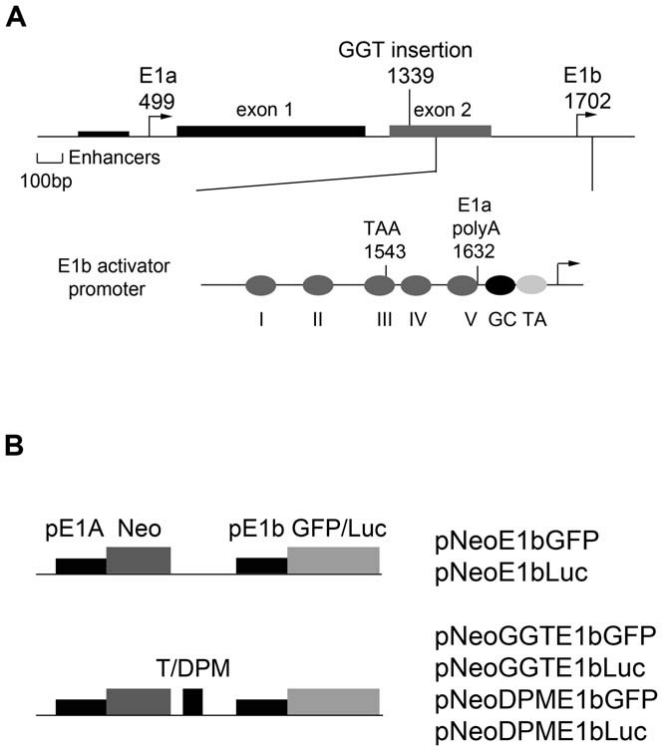
Genetic system for analysis of read-through activation. A. Organization of the region of the adenovirus 5 genome to the left of the E1b transcription start. The top diagram shows the relationship of the two major transcription initiation sites (E1a, nucleotide position 499; and E1b, 1702) and the position of insertion of the ectopic translation termination sequence *GGT* (1339). The E1a enhancer region is indicated as a filled rectangle and alternate E1a transcription initiation sites as arrows. The E1a coding exons are shaded (exon 1) and open (exon 2) rectangles. Below is an expanded view of the E1b transcriptional control region indicating mapped sites of DNA-protein interaction in the distal region (I-V), and proximal binding sites for Sp1 (GC) and TBP (AT). The locations of the E1a translation termination codon and poly(A) addition site within the E1b control region also are indicated. B. Selection marker-reporter plasmids: structures of plasmids with *neo* and *gfp* or *luc* gene replacements. pE1a: E1a promoter; Neo: G418 resistance gene; T: termination sequences, *GGT* or *DPM*; pE1b: E1b promoter; GFP: green fluorescent protein gene; Luc: luciferase gene.

Transcription from an upstream promoter also can activate a downstream promoter. The E1a and E1b genes of adenovirus 5 are tightly linked. Part of E1a exon 2, including amino acid coding sequences and the 3′ untranslated region, overlaps the E1b promoter region [13]–[15] (Figure 1A). Primary transcripts initiated from the E1a promoter invade the E1b promoter and coding region, and these read-through transcripts are processed to produce E1a mRNA [16], [17]. Only transcripts that originate from the E1b promoter are precursors of E1b mRNA [18], [19]. Artificial termination of read-through transcription from the E1a promoter by insertion of ectopic transcription termination sequences (*GGT*: globin gene termination sequence) dramatically reduces early E1b gene expression *in cis* [20]–[22]. Since point mutations that inactivate the transcription termination function of *GGT* restore downstream promoter activity, the ability to block read-through transcription is the only property of *GGT* that is required for inhibition [21], [22]. Therefore, read-through transcription is required for wild-type (wt) activity of the E1b promoter early after infection. There is evidence that the mechanism of the interaction depends upon a *cis*-dominant property of the early viral template [21] but the effect is “local” rather than global [22].

The mechanism of read-through activation could depend on the structure of early adenovirus nucleoprotein, which is likely to be different from that of cellular chromatin. Alternatively, read-through activation might not require early viral chromosome structure, allowing adjacent genes to take advantage of this interaction for coordinated expression in the context of cellular chromatin. To explore this possibility, read-through activation from reporter constructs that retained the basic genetic organization of the E1a-E1b region from the viral genome was evaluated in the context of templates stably integrated into the cellular genome.

## Results

Read-through activation early after infection is readily observed with recombinant adenoviruses that have the E1b coding region replaced by the *luc* reporter gene [22]. In these viruses, insertion of the mouse β-globin gene transcription termination sequence *GGT* between E1a and E1b strongly reduces early expression of the downstream *E1b-luc* gene, whereas inactivation of the termination function of the inserted sequence restores E1b promoter function.

To determine whether read-through activation affected gene expression in chromosomal DNA copies stably integrated in the cellular genome, we constructed plasmids with regulatory sequence arrangements identical to those in the virus but with coding region gene replacements to facilitate selection and analysis of cell lines. The E1a coding region was replaced with a selectable marker gene (*neo*, G418 resistance) and the *gfp* or *luc* reporter genes were substituted for the E1b coding region. This strategy allowed read-through activation in G418-resistant cell lines to be scored readily by evaluating fluorescence intensity or luciferase production. To evaluate the read-through requirement for reporter gene expression, plasmids contained *GGT* (pNeoGGTE1bGFP and pNeoGGTE1bLuc), *GGT* inactivated by a double set of point mutations (*DPM*, pNeoDPME1bGFP and pNeoDPME1bLuc), or no insertion (pNeoE1bGFP and pNeoE1bLuc) between the *E1a-neo* and *E1b-*reporter genes (Fig. 1B).

We considered targeting insertions to a specific site for these experiments. However, whether any particular site contained *cis*-dominant elements or properties that might affect read-through activation could not be predicted. On the other hand, it was likely that the potential for *cis*-dominant effects of the integration site to confound the analysis would be revealed by comparing the results from either pooled or individual cell clones with untargeted integration sites. Accordingly, untargeted clones were isolated and both pooled and individual clones were analyzed. Also, it was possible that *cis*-acting effects of a particular selection or reporter gene would interfere with read-through activation. To reduce the possibility that such interference would compromise the analysis, the plan was to use two selection genes, as well as the two reporter genes. However, attempts to isolate puromycin-resistant HeLa cells from E1a promoter-driven constructs analogous to the plasmids shown in Fig. 1B were unsuccessful (data not shown).

**Figure 2:**
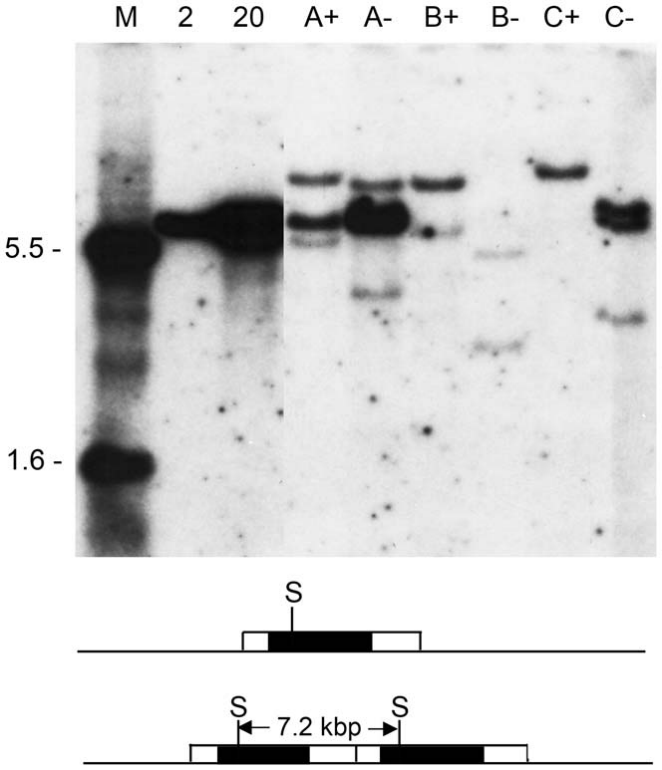
Integrated NeoGGTE1bGFP sequences in stable cell lines. DNA was extracted from G418-resistant stable cell lines established with the plasmid pNeoGGTE1bGFP (Fig. 1b). Blots were prepared and probed as described in Materials and Methods. Lane designations: M, size markers derived from an *Eco*RI/*Psh*A1 digest of the plasmid (sizes shown on the left); 2, reconstruction with 2 copies of *Sph*I-digested (linear) plasmid (7.2 kbp), based on quasi-tetraploid human DNA content; 20, reconstruction with 20 copies of plasmid; A+, GFP-expressing cell line 1 isolated after electroporation of 1 µg of DNA; A−, GFP-negative cell line from the same experiment as A+; B+, GFP-expressing cell line isolated in a second experiment after electroporation of 1 µg of DNA; B−, GFP-negative cell line from the same experiment as B+; C+, GFP-expressing cell line isolated after lipofection with 1 µg of DNA; C−, GFP-negative cell line 2 from the same experiment as C+. An irrelevant lane was removed from between lanes 20 and A+. The diagram below shows the results expected from a single (top) or tandem (bottom) integration of the complete plasmid sequence (open box). For the former, the sizes of the two junction DNA fragments that hybridize to the probe (shaded box) will depend on the site of insertion. For the latter, in addition to the junction fragments, a unit length band (7.2 kbp) will be generated and its intensity will depend on the number of copies in the tandem array. There is a single site for *Sph*I (S) cleavage in the plasmid *neo* gene.

To obtain integrated sequence arrangements in the untargeted clones as similar as possible to that of the E1 gene cluster in the adenovirus genome, we sought conditions for DNA introduction predicted to favor low copy number integrations. For electroporated DNA, parameters can be adjusted to produce integration of from one to about 20 copies of plasmid DNA [23]–[26], as opposed to a large amount of genomic DNA in each cell [27]. Similar information is not available for lipofection. For three different experiments, companion G418-resistant cell lines expressing high (+) or undetectable (−) levels of GFP after introduction of the pNeoGGTE1bGFP plasmid were analyzed for the pattern of integrated plasmid DNA. The results (Fig. 2) show that we were successful in obtaining cells lines with low copy number integrations. None of the cell lines had more than a few copies of integrated plasmid. There was no relationship between GFP expression and a particular integration pattern. GFP-expressing cell lines had both single copy (B+, C+) and tandem copy (A+) insertions. Likewise, GFP-negative lines were single copy (B−) or multi-copy (A−, C−). Cell line B- may not have retained a viable copy of the GFP gene. Also, at least in this small sample, there seemed to be no advantage to be gained by selecting clones of a particular integration class to standardize the subsequent analysis.

**Figure 3:**
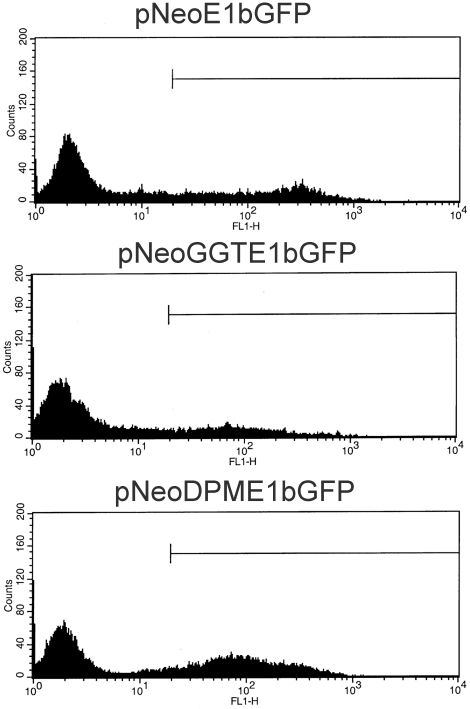
Histograms of GFP-expression in cells from pooled colonies analyzed by flow cytometry. The plots from Experiment 2 of Table 2 show the gating for the threshold of GFP-positive designation. The plasmid used to generate the colonies is indicated above each histogram.

**Figure 4:**
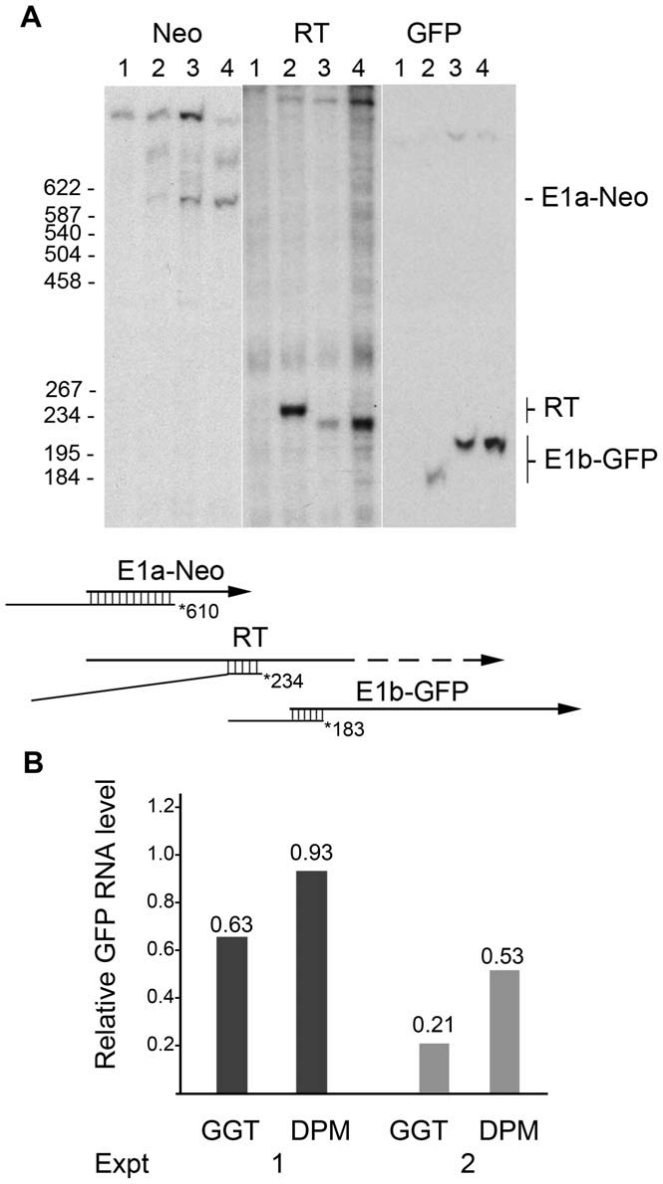
Read-through activation in Neo-GFP cell lines. A. Nuclear RNA prepared from pooled cultures of cell lines isolated after electroporation with 1 µg of DNA was assayed by hybridization and protection from nuclease S1 digestion. Expression of *E1a-neo,* read-through transcription (RT), and *E1b-gfp* produced the specific protected bands indicated in the diagram below the autoradiographic data. The arrows indicate the relative positions of the transcripts in the template (the uncertain end of the read-through transcript is indicated by the dashed line). The probe is indicated by a line with the position of the 5′-end label shown as an asterisk. The region of the probe protected by each transcript is indicated as double-stranded (DNA-RNA hybrid). The position of divergence of the sequence of the probe for read-through transcription from the read-through RNA product is shown by the loss of DNA-RNA hybrid formation. Variation in migration of the RT and E1a-GFP products probably was caused by sequence differences at the junction site produced during plasmid construction. Lane designations: 1: HeLa cells; 2: culture derived from pNeoE1bGFP; 3: culture derived from pNeoGGTE1bGFP; 4: culture derived from pNeoDPME1bGFP. The positions of size markers (not shown) are indicated on the left of the autoradiograms. B. *E1b-GFP* RNA levels from two experiments (Expt 1 is shown in A) were quantified and normalized to the quantity of *E1a-Neo* RNA. The results are expressed relative to the pNeoE1bGFP value (1.00).

In the first set of experiments, GFP expression was evaluated quantitatively either by visual scoring of individual G418-resistant colonies (Table 1) or flow cytometry of pooled cell cultures (Table 2, representative histograms of GFP-expressing cells in the cultures are shown in Fig. 3). For the latter, G418 was omitted during the plating to avoid selective amplification of any of the original clones and to maintain the GFP profile of the population. By either method, GFP expression was inhibited by insertion of *GGT* and expression was restored by inactivation of the transcription termination sequence (*DPM*, Tables 1 and 2, Fig. 3). Similar results were obtained when RNA was assayed in the pooled cultures by hybridization and nuclease protection (Fig. 4). This method allowed correctly initiated transcripts to be measured, a particularly important consideration for *E1b*-promoted RNA since read-through transcripts initiated from the E1a promoter can contain E1b sequences [e.g., [(17)]]. Standardized to the amounts of *E1a-neo* RNA, *E1b-gfp* transcription from the integrated DNA was inhibited by *GGT* and partially restored when the terminator was inactivated (Fig 4A, “Neo” and “GFP” panels, quantification shown in Fig. 4B). As observed previously in early virus infections, *GGT* reduced read-through transcription substantially and inactivation of the termination function partially restored levels of read-through transcription (Fig. 4A, “RT” panel). These results show that termination of *E1a-neo* transcription interfered *in cis* with expression of the downstream *E1b-gfp* gene from integrated DNA copies.

**Table 1:** Effect of *GGT* insertion on the expression of a downstream *gfp* reporter in G418-resistant cell lines.

DNA Preparation	µg DNA added	Transfection procedure	% colonies expressing GFP *(total No. scored)*		
			No GGT^a^	GGT	DPM^b^
1	1	Lipofection	67 *(141)*	54 *(169)*	69 *(123)*
	5	Electroporation	49 *(49)*	35 *(111)*	50 *(62)*
	5	Electroporation	27 *(41)*	20 *(41)*	40 *(102*
	2	Electroporation	24 *(79)*	11 *(291)*	16 *(146)*
	1	Electroporation	28 *(25)*	3.1 *(97)*	29 *(42)*
	1	Electroporation	50 *(39)*	25 *(84)*	62 *(93)*
2	1	Electroporation	21 *(168)*	13 *(271)*	21 *(68)*
	1	Electroporation	19 *(128)*	16 *(238)*	39 *(147)*
	1	Electroporation	35 *(83)*	18 *(96)*	39 *(104)*

a difference from GGT, p<0.0003, paired t-test.

b difference from GGT, p<0.0003, paired t-test.

**Table 2:** Effect of GGT insertion on GFP expression in pooled cultures of G418-resistant colonies^a^.

Plasmid	Experiment No.			
	1		2	
	%fluorescent^b^	meanintensity^c^	%fluorescent	meanintensity
pNeoE1b GFP	45	470	31	73
pNeoGGTE1bGFP	28	146	21	30
pNeoDPME1bGFP	61	442	44	64
None	0.0	3.4	nd^d^	

a Colonies (collected from three plates per plasmid) in pooled cultures were harvested and analyzed by flow cytometry as described in Materials and Methods.

b percentage of cells in the population with FL1-H>10^1.1^.

c per cell.

d not done.

**Figure 5:**
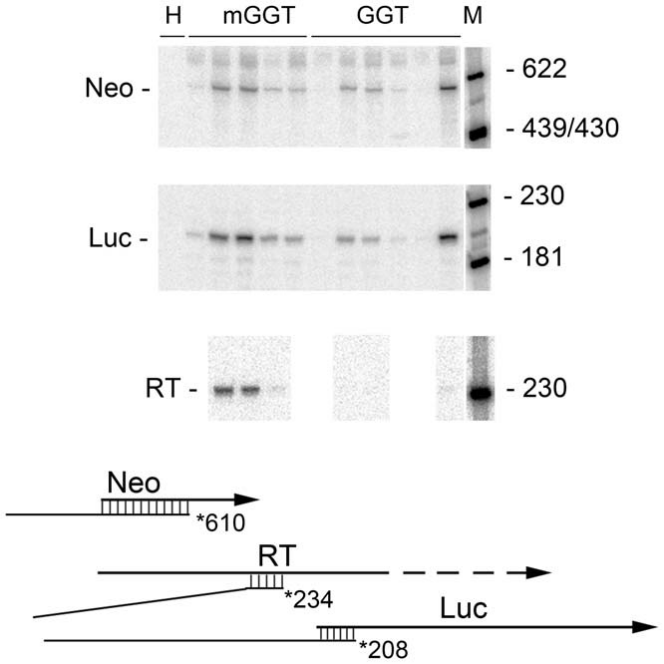
Read-through activation in Neo-Luc cell lines. Total RNA prepared from individual cell lines isolated after electroporation with 1 µg of DNA was assayed by hybridization and protection from nuclease S1 digestion. Expression of *E1a-neo,* read-through transcription (RT), and *E1b-luc* produced the specific protected bands indicated in the diagram below the autoradiographic data. The diagram is laid out as in Fig. 4. Each lane represents a different cell line. He: Hela cells; DPM: cell lines derived from pNeoDPME1bLuc; GGT: cell lines derived from pNeoGGTE1bLuc; M: size standards.

Individual G418-resistant clones produced by transfection with NeoLuc plasmids were screened for luciferase activity and luciferase-positive clones derived from the different plasmids were analyzed quantitatively for RNA production by hybridization protection (Fig. 5). The cell lines differed markedly in the transcription activity of the NeoLuc cassette. *E1a-neo* and *E1b-luc* RNA levels (Table 3) varied more than thirty-fold and the latter correlated well with the results of luciferase assays from the same cell line (data not shown). After normalization to *E1a-neo* RNA, *E1b-Luc* RNA expression in the cell lines containing an inactivated termination sequence (DPM) was increased over the levels obtained from inserted sequences with a functional terminator (GGT) (Table 3). As with the NeoGFP cassettes, the termination sequence was effective in reducing read-through transcription to near background levels in all of the cell lines obtained by insertion of a NeoGGTE1bLuc expression unit (Table 3). Note that some cell lines produced levels of read-through RNA that were below the threshold that could be measured (nd, Table 3). These results support the conclusion obtained with the NeoGFP cell lines, that integrated DNA can support read-through activation.

**Table 3:** Effect of read-through transcription on E1b-Luc RNA expression in stable cell lines^a^.

Cell line	E1a-Neo RNA	E1b-Luc RNA	Ratio^b^	RT RNA^c^
GGT1	2.4	1.6	0.67	nd^d^
GGT2	18.9	18.2	0.96	0.5
GGT4	16.3	12.4	0.76	0.7
GGT5	5.4	4.0	0.74	nd
GGT10	2.3	2.6	1.13	nd
GGT11	53.7	60.3	1.12	0.5
DPM2	5.7	7.9	1.38	nd
DPM3	32.7	39.5	1.21	7.1
DPM6	30.8	50.4	1.64	7.0
DPM10	10.9	18.7	1.71	2.8
DPM11	13.1	15.0	1.15	nd

a Monolayers at 70-90% confluence were harvested and the RNAs indicated were assayed and quantified from the experiment shown in Fig. 4. All units are arbitrary.

b E1a-Luc/E1a-Neo; mean +/− st. dev: GGT cell lines: 0.90+/−0.20; DPM cell lines: 1.42+/−0.25; difference between GGT and DPM, p<0.006, unpaired t-test.

c Normalized to E1a-Neo.

d Not determined.

## Discussion

Here we provide evidence that cellular chromatin supports read-through activation of a closely linked gene. The activation of downstream gene expression was observed at both population and individual clone levels in non-targeted gene insertions. Read-through transcription enhances expression of a downstream HIV-1 proviral genome inserted into an intron of the dihydrofolate reductase (DHFR) gene when the DHFR promoter and HIV promoter are in the same orientation [28]. Our results support and extend this finding by showing that activation by read-through transcription in the cellular genomic context did not require either a specific site of integration or the retrovirus proviral elements. Accordingly, the read-through activation mechanism should be available to coordinate gene expression of closely linked genes in both viral and cellular genomes. Non-coding transcription also has been implicated in transcriptional activation of at least one downstream cellular gene [29], although a *trans*-acting function for the non-coding transcript was not ruled out completely.

Activation in *cis* of downstream gene expression in the cells lines by read-through transcription was modest, about two-fold in our experimental system, and less than the magnitude of activation we observed in early virus infections [21], [22]. The modest effect of read-through on activation of the downstream reporter in cell lines could be related to the use of the Neo gene for selection. Silencing of linked promoters by *neo* was reported previously in assays of activity from transiently transfected templates and integrated sequences [30]. We also observed sharply reduced *E1a-neo* and *E1b*-mediated transcription in adenovirus strains with Neo gene replacements for E1a (D. Spector, unpublished data). Conceivably, the silencing elements in the Neo coding region, or other DNA sequences in that region, could directly impact the activation mechanism as well. If so, identification of the relevant sequences in the Neo coding region could provide further insight into the mechanism of activation.

Gene expression from integrated sequences also is undoubtedly affected by the local chromatin environment and nearby regulatory elements. In fact, selection for Neo expression probably favors the survival of cell lines with integration sites in genomic locations that are transcriptionally active. Such a bias might increase the probability of integration in the vicinity of a strong cellular enhancer; strong enhancers relieve the requirement for activation by read-through transcription in adenovirus [22]. If the same is true for the cellular copies integrated near strong enhancers, then *GGT*-containing clones should have higher levels of *E1b*-mediated expression than expected. This circumstance would also result in underestimation of the potential benefit from the read-through mechanism.

We note that, as in viruses, *GGT* blocked read-through transcription in integrated sequences and that mutation of the hexanucleotide recognition sequence for polyadenylation restored read-through. These results highlight the versatility of the β-globin element as an effective transcription termination sequence.

We analyzed read-through from randomly integrated expression cassettes because we could not predict how any particular configuration of the targeted integration site would affect the interaction. The results here provide some guidance in that respect. It would seem desirable to minimize potential effects of nearby control elements, either by utilizing an “inert” site or providing insulator sequences [31], [32] as boundaries for the expression cassette. Also, careful consideration must be given to choice of selectable marker and reporter gene sequences to ensure that they are inert as well with respect to the molecular mechanism under investigation.

## Materials and Methods

### Cell culture

HeLa cells (ATCC-CCL-2) were purchased from Flow Laboratories (currently MP Biomedicals). Monolayers were maintained in DMEM with 5% fetal bovine serum as described previously [13], [21].

### Recombinant DNA

Plasmids with neomycin (Neo) resistance gene (*neo*) and the green fluorescent protein (GFP) reporter gene (*gfp)* were constructed from two sources. The Neo and GFP genes were derived from the donor plasmid, pEGFP-N3 (Clontech). The genes were inserted into “term” vector plasmids, which contain E1a enhancer-promoter regulatory sequences, a wt or mutated *GGT*, respectively, and the E1b promoter and gene.

The backbones of the “term” vectors were plasmids pm563, which has a mutation at the 5′ end of the E1a coding region that introduces an *Nco*I site [15], [33], and p563/112 [15]. The former contains adenovirus sequences from E1a and the 5′ portion of E1b, whereas the latter includes most of the E1b gene and has an E1b *dl*112 allele [34]. pm563Δ lacks E1a sequences between positions 563 and 1338, which were removed by excising the DNA between the unique *Nco*I and *Xba*I sites in the plasmid and adding an *Xba*I linker to retain the *Xba*I site at the junction. To make the first donor “term” plasmid, pΔterm, the *Msc*I-*Bgl*II fragment of pβmajG [obtained from Erik Falck-Pedersen [20]], containing the mouse β^maj^ gene transcription termination sequence, was inserted into the *Xba*I site of pm563Δ.

The second donor “term” plasmid, pΔtermdpm, was constructed in multiple steps. First, pterm and pterm112 were constructed by inserting the *Msc*I-*Bgl*II fragment of pβmajG into the *Nco*I site of pm563 or p563/112, respectively. Next, pΔterm112 was produced by exchanging the *Hpa*I fragment from pΔterm that includes the E1a deletion for that of pterm112. Then, the *Bst*XI- *Xba*I fragment in pΔterm112 that contains the polyadenylation sites in the transcription termination sequence was exchanged for the corresponding fragment from pD''EF (obtained from Erik Falck-Pedersen [20]), which has those sites inactivated by mutation, to produce pΔtermdpm112. The deleted E1a region was restored by exchange of the *Hpa*I fragment from pterm to produce plasmid ptermdpm112. Finally, the *Eco*RI-*Xba*I fragment from ptermdpm112 that contains the mutated polyadenylation sites was exchanged into pΔterm to produce pΔtermdpm.

To produce the plasmids containing *neo* and *gfp*, a fragment containing the Neo coding region was excised from pEGFP-N3 by *Avr*II digestion and inserted into the *Bse*R1 site of pΔterm or pΔtermdpm. The resulting plasmids, with *neo* in place of the E1a coding region, were designated pNeoGGT and pNeoDPM. A *Sma*I-*Ssp*I fragment containing the GFP coding region, its polyadenylation signals, and polyadenylation site, was excised from pEGFP-N3 and substituted for an *Eco*NI-*Hin*dIII fragment (the E1b gene region) of the NeoGGT and NeoDPM plasmids. The resulting plasmids, pNeoGGTE1bGFP and pNeoDPME1bGFP, contained *neo* driven by the E1a enhancer-promoter (*E1a-neo*), a wt or mutated *GGT* terminator, and *gfp* under the control of the E1b promoter (*E1b-gfp*).

A third plasmid, pNeoE1bGFP, without termination sequences between the two genes was constructed by excising the *CT* termination sequence [22], as a *Bst*XI-*Hind*III fragment, from pNeoCTE1bGFP and self-ligating the vector. To construct pNeoCTE1bGFP, a *Bst*XI-*Sal*I fragment that contains *CT* and the E1b promoter was excised from pACCTE1bLuc [22] and substituted for a *Bst*XI-*Bam*HI fragment, containing *GGT* and the E1b promoter, of pNeoGGTE1bGFP.

The expression cassettes from pNeoE1bGFP, pNeoGGTE1bGFP, or pNeoDPME1bGFP were excised as *Eco*RI-*Nde*I fragments and substituted for the *Eco*RI-*Fse*I fragment in pAC343CTE1bLuc [22], which includes the E1a and *E1b-*luciferase gene *(luc)* regions, to produce pACNeoE1bGFP, pACNeoGGTE1bGFP, or pACNeoDPME1bGFP, respectively. Plasmids with *neo* in place of E1a and *luc* in place of E1b were constructed by exchanging a *Psh*AI-*Eco*RI fragment of p343E1bLuc [22] containing *E1b-luc* for a similar fragment containing *E1b-gfp* in pACNeoE1bGFP, pACNeoGGTE1bGFP, or pACNeoDPME1bGFP. The resulting plasmids were designated pNeoE1bLuc, pNeoGGTE1bLuc, or pNeoDPME1bLuc, respectively.

### Stable cell line isolation

Both lipofection and electroporation were used for DNA uptake and isolation of stable neomycin-resistant cell lines with copies of plasmid DNA randomly integrated into the cellular genome. For lipofection, HeLa cells were subcultured into 6-well plates one day before transfection at 70–90% of confluence. 3 µl of Fugene 6 reagent and 1 µg of plasmid DNA were diluted to a volume of 100 µl with Opti-MEM medium and added to 1 well. After 48 hrs, cells were subcultured 1 to 30 into 150 mm dishes and placed under G418 selection (400 µg/ml) for two weeks until resistant cell colonies appeared.

For electroporation, HeLa cells were removed from monolayer surfaces and suspended in electroporation buffer (1× HeBSS, pH 7.10) at a concentration of 1×10^7^ cells/ml. 1 ml of cell suspension was transferred to a sterile electroporation cuvette. Linearized plasmid DNA (1, 2, or 5 µg) was added to the cuvette and the suspension was mixed by inversion. Electroporation was performed at a setting of 230V/960 µF in the Gene Pulser XcellTM (Bio-Rad Laboratories, Inc.). The suspension was removed and sequential rinses were performed with 0.8 ml aliquots of medium to ensure complete removal of the cells. The rinses were combined with the electroporated cells and transferred to a T75 flask containing 40 ml of medium. After allowing 48 hrs for cell recovery, the cells were inoculated into 150 mm dishes at 6×10^5^ cells/dish, corresponding to three to six dishes for each electroporated cell sample and construct. Cells were placed under G418 selection (400 µg/ml) for two weeks until resistant colonies appeared. The *E1b-luc*-containing colonies were trypsinized and subcultured into 12-well plates (one colony per well). After reaching confluence, cells from a single well were expanded for maintenance under neomycin selection and screened for luciferase activity. About 70% of the neo-resistant colonies produced measurable luciferase.

### RNA analysis

Total RNA was prepared from cell lines with Trizol (Invitrogen Corp.) using the protocol provided by the supplier. Specific transcripts were quantified by hybridization and protection from nuclease S_1_ digestion as described previously [15]. For hybridization, double-stranded probes labeled at a single 5′-end with T4 polynucleotide kinase and [gamma-^32^P] ATP were prepared as described previously [15]. To detect *E1a-neo* transcription, an 1126-bp probe was prepared as a *Nco*I (labeled site)-*Eco*RI fragment from pNeoGGTE1bGFP or pNeoE1bLuc. To detect *E1b-luc* RNA, a 1576-bp probe was prepared as a *Sca*I (labeled site)-*Sac*II fragment of pAC343E1bLuc [22]. To detect *E1b-gfp* transcription, a 536-bp probe was isolated as a *Bss*SI (labeled site)–*Psh*AI fragment from pNeoGGTE1bGFP. To detect read-through transcription from cell lines with the *luc* gene, a 1215-bp probe was prepared as a *Hpa*I (labeled site)-*Sac*II fragment of pAC343E1bLuc. To detect read-through transcription from cell lines with *E1b-gfp* genes, a 1410-bp probe was prepared as an *Hpa*I-*Fsp*I fragment from pNeoCTE1bGFP or pNeoCTDPME1bGFP. The latter was constructed by isolating a *Bst*XI-*Sal*I fragment from pACCTdpmE1bLuc [22] and replacing the *Bst*XI-*Bam*HI fragment of pNeoGGTE1bGFP.

Band intensities were quantified at the Penn State Hershey Core Facility by scanning densitometry of ×-ray film (Kodak ×AR5) in a GS-800 Calibrated Densitometer (Bio-Rad), or by using a Molecular Dynamics phosphorimaging screen that was analyzed with Quantity One software and an FX scanner (Bio-Rad Laboratories, Inc.). E1b-dependent transcription was normalized for template copy number and gel loading as described previously [15].

### Luciferase assays

Luciferase assays were performed on cell lines harvested at about 80% confluence. Cell lysates were prepared and the luciferase activity was assayed using the Luciferase Assay System with Reporter Lysis Buffer (Promega Corp.) according to the manufacturer's protocol. Light emission was quantified on an FB12 Luminometer (Zylux Corp.). All values were normalized to extract protein concentrations (DC protein assay; Bio-Rad Corp.).

### Flow cytometry analysis

Flow cytometry was performed at the Penn State Hershey Core Facility. For determination of GFP expression, G418-resistant colonies generated as described above were trypsinized, pooled, and the cells were replated for expansion without G418 selection. Cell suspensions were prepared subsequently at a concentration of about 1×10^6^ cells/ml and about 10,000 cells were analyzed in each flow cytometry run. Cells with a fluorescence intensity parameter FL1-H ≥10^1.1^ (determined from a sample that expressed no GFP) were scored. The percentage of green cells in the population and the fluorescence intensity per cell were recorded for each sample. Values from replicates were averaged.

### DNA blot hybridization analysis

To detect plasmid DNA sequences integrated in the genomes of stable cell lines, G418-resistant colonies obtained as described above were trypsinized and subcultured into 6-well plates (1 colony per well). After reaching confluence, cells from a single well were expanded for maintenance under neomycin selection. Cellular DNA was extracted and digested (10 µg/sample) with *Sph*I, and blot hybridization was performed as described [35]. The probe was an *Eco*RI-*Nde*I fragment that contains the full expression cassette in plasmid pNeoE1bGFP. Reconstructions were performed with salmon sperm DNA but calculations were based on copies per quasi-tetraploid human DNA content of HeLa cells.

## References

[1] Fedoroff NV (1979). On spacers.. Cell.

[2] Tuan D, Solomon W, Li Q, London M (1985). The β-like globin gene domain in human erythroid cells.. Proc Natl Acad Sci USA.

[3] Orkin SH (1990). Globin gene regulation and switching: circa 1990.. Cell.

[4] Moss T (1983). A transcriptional function for the repetitive ribosomal spacer in Xenopus laevis.. Nature.

[5] Cassidy BG, Yang-Yen HF, Rothblum LI (1987). Additional RNA polymerase I initiation sites within the nontranscribed spacer region of the rat rRNA gene.. Mol Cell Biol.

[6] Grimaldi G, Fiorentini P, diNocera PP (1990). Spacer promoters are orientation-dependent activators of pre-rRNA transcription in Drosophila melanogaster.. Mol Cell Biol.

[7] Paalman MH, Henderson SL, Sollner-Webb B (1995). Stimulation of the mouse rRNA gene promoter by a distal spacer promoter.. Mol Cell Biol.

[8] Kong S, Bohl D, Li C, Tuan D (1997). Transcription of the HS2 enhancer toward a cis-linked gene is independent of the orientation, position, and distance of the enhancer relative to the gene.. Mol Cel Biol.

[9] Bateman E, Paule M (1988). Promoter occlusion during ribosomal RNA transcription.. Cell.

[10] Cook W, Wobbe K, Boni J, Coen D (1996). Regulation of neighboring gene expression by the herpes simplex virus type 1 thymidine kinase gene.. Virology.

[11] Corbin V, Maniatis T (1989). Role of transcriptional interference in the Drosophila melanogaster adh promoter switch.. Nature.

[12] Esperet C, Sabatier S, Devile M, Ouazana R, Bouhassira E (2000). Non-erythroid genes inserted on either side of human HS-40 impair the activation of its natural alpha-globin gene targets without being themselves preferentially activated.. J Biol Chem.

[13] Parks CL, Banerjee S, Spector DJ (1988). Organization of the transcriptional control region of the E1b gene of adenovirus type 5.. J Virol.

[14] Parks CL, Spector DJ (1990). cis-Dominant defect in activation of adenovirus type 5 E1b early RNA synthesis.. J Virol.

[15] Spector DJ, Parks CL, Knittle RA (1993). A multicomponent cis-activator of transcription of the E1b gene of adenovirus type-5.. Virology.

[16] Thomas GP, Mathews MB (1980). DNA replication and the early to late transition in adenovirus infection.. Cell.

[17] Montell C, Fisher EF, Caruthers MH, Berk AJ (1983). Inhibition of RNA cleavage but not polyadenylation by a point mutation in mRNA 3′ consensus sequence AAUAAA.. Nature.

[18] Wilson MC, Darnell JE (1981). Control of messenger RNA concentration by differential cytoplasmic half life. Adenovirus messenger RNAs from transcription units 1A and 1B.. J Mol Biol.

[19] Sussenbach JS, Ginsberg HS (1984). The structure of the genome.

[20] Falck-Pedersen E, Logan J, Shenk T, Darnell JE (1985). Transcription termination within the E1a gene of adenovirus induced by insertion of the mouse β-major globin termination element.. Cell.

[21] Maxfield LF, Spector DJ (1997). Readthrough activation of early adenovirus E1b gene transcription.. J Virol.

[22] Shen L, Spector DJ (2003). Local character of readthrough activation in adenovirus type 5 early region 1 transcription control.. J Virol.

[23] Potter H, Weir L, Leder P (1984). Enhancer-dependent expression of human kappa immunoglobulin genes introduced into mouse pre-B lymphocytes by electroporation.. Proc Natl Acad Sci USA.

[24] Toneguzzo F, Hayday AC, Keating A (1986). Electric field-mediated DNA transfer: transient and stable gene expression in human and mouse lymphoid cells.. Mol Cell Biol.

[25] Toneguzzo F, Keating A (1986). Stable expression of selectable genes introduced into human hematopoietic stem cells by electric field-mediated DNA transfer.. Proc Natl Acad Sci U S A.

[26] Toneguzzo F, Keating A, Glynn S, McDonald K (1988). Electric field-mediated gene transfer: characterization of DNA transfer and patterns of integration in lymphoid cells.. Nucleic Acids Res.

[27] Jastreboff MM, Ito E, Bertino JR, Narayanan R (1987). Use of electroporation for high-molecular-weight DNA-mediated gene transfer.. Exp Cell Res.

[28] Han Y, Lin YB, An W, Xu J, Yang H-C (2008). Orientation-dependent regulation of integrated HIV-1 expression by host gene transcriptional readthrough.. Cell Host & Microbe.

[29] Abarrategui I, Krangel MS (2007). Noncoding transcription controls downstream promoters to regulate T-cell receptor alpha recombination.. EMBO J.

[30] Artelt P, Grannemann R, Stocking C, Friel J, Bartsch J (1991). The prokaryotic neomycin-resistance-encoding gene acts as a transcriptional silencer in eukaryotic cells.. Gene.

[31] Emery DW, Yannaki E, Tubb J, Stamatoyannopoulos G (2000). A chromatin insulator protects retrovirus vectors from chromosomal position effects.. Proc Natl Acad Sci U S A.

[32] Steinwaerder DS, Lieber A (2000). Insulation from viral transcriptional regulatory elements improves inducible transgene expression from adenovirus vectors in vitro and in vivo.. Gene Ther.

[33] Whyte P, Ruley HE, Harlow E (1988). Two regions of the adenovirus early region 1A proteins are required for transcription.. J Virol.

[34] Babiss LE, Fisher PB, Ginsberg HS (1984). Effect on transformation of mutations in the early region 1b-encoded 21- and 55-kilodalton proteins of adenovirus 5.. J Virol.

[35] Spector DJ (1983). The pattern of integration of viral DNA sequences in the adenovirus 5-transformed human cell line 293.. Virology.

